# Reimagining the role of the nursing workforce in Uganda after more than a decade of ART scale-up

**DOI:** 10.1186/s12960-020-00479-7

**Published:** 2020-05-29

**Authors:** Henry Zakumumpa

**Affiliations:** Makerere University, School of Public Health, P O Box 7072, Kampala, Uganda

**Keywords:** Health systems, HIV, Nurses, Task shifting, Differentiated service delivery

## Abstract

**Background:**

The expanding roles and increasing importance of the nursing workforce in health services delivery in resource-limited settings is not adequately documented and sufficiently recognized in the current literature. Drawing upon the theme of 2020 as the International Year of the Nurse and the Midwife, we set out to describe how the role of nurses expanded tremendously in health facilities in Uganda during the era of anti-retroviral therapy (ART) scale-up that commenced in June 2004.

**Methods:**

We employed a mixed-methods sequential explanatory research design. Phase I entailed a cross-sectional health facility survey (*n* = 195) to assess the extent to which human resource management strategies (such as task shifting) were common. Phase II entailed a qualitative multiple case study of 16 (of the 195) health facilities for an in-depth understanding of the strategies adopted (e.g. nurse-centred HIV care). Descriptive analyses were performed in STATA (v 13) while qualitative data were analysed by thematic approach.

**Results:**

We found that nurses were the most represented cadre of health workers involved in the overall leadership of HIV clinics across Uganda. Most nurse-led HIV clinics were based in rural settings; however, this trend was fairly even across setting (rural/urban/peri-urban). While 181 (93%) health facilities allowed non-physician cadre to prescribe ART, a number of health facilities (*n* = 36) or 18% deliberately adopted *nurse-led* HIV care models. Nurses were empowered to be multi-skilled with a wide range of competencies across the HIV care continuum right from HIV testing to mainstream clinical HIV disease management. In several facilities, nursing cadre were the backbone of ART service delivery. A select number of facilities devised differentiated models of task shifting from physicians to nurses in which the latter handled patients who were stable on ART.

**Conclusion:**

Overall, our study reveals a wide expansion in the scope-of-practice of nurses during ART scale-up in Uganda. Nurses were thrust in roles of HIV disease management that were traditionally the preserve of physicians. Our study underscores the importance of reforming regulatory frameworks governing nursing workforce scope of practice such as the need for developing a policy on task shifting which is currently lacking in Uganda.

## Background

Sub-Saharan Africa is faced with a severe human resources for health crisis despite having an overwhelming disease burden [1–3]. The majority of the 57 countries listed by the World Health Organization (WHO) as having a human resources for health crisis are from this region alone [4]. Part of this crisis is manifested in the acute shortage of health workers across multiple cadre and at all levels of the health system [5]. The shortage of physicians is an especially pronounced constraint which is compounded by labour market dynamics that render many public facilities unable to attract and retain physicians [5]. As such, countries in sub-Saharan Africa have grown to increasingly rely on nurses or other non-physician cadre especially those with shorter tertiary training cycles or those who are less-specialized [6]. In many countries in sub-Saharan Africa, such as Uganda, nurses constitute as high as 80% of the entire health workforce [7].

The HIV and AIDS epidemic exacerbated these staffing shortages and dramatically increased workloads due to escalating HIV client loads across sub-Saharan Africa [3]. Nurses were thrust into roles they were not prepared for by virtue of their pre-service training considering that ‘nursing education mainly focuses on clinical skills and theory related to patient care and management’ [8]. Nurses were brought to the frontlines of national HIV epidemic responses and drafted into a range of clinical HIV services which were traditionally the preserve of physicians based on western models of care that derive from better resourced health systems [9]. However, the continuous increase in demand for HIV treatment brought on, partly, by the lowering of thresholds of eligibility for treatment over the past two decades demanded pragmatic shifts [10]. The World Health Organization (WHO) released policy guidelines recommending task shifting from physicians to non-physicians such as nurses and midwives ‘the WHO recognizes that nurses and midwives can provide services in the clinical setting, such as HIV clinical staging and the management of opportunistic infections’ [11]. Although several countries are yet to formally adopt task shifting of clinical HIV disease management from physicians to nurses in their regulatory frameworks [12, 13], some countries have. In 2010, South Africa released policy guidelines providing for Nurse-Initiated and Managed Antiretroviral Therapy (NIMART) [14]. This was one of the watershed moments in the re-imagining of the role of the nursing workforce in health services delivery in general. There are several studies demonstrating the non-inferiority of nurse-managed HIV care and treatment [15–19].

### HIV treatment in Uganda prior to the era of ART scale-up

Before the advent of antiretroviral therapy (ART) scale-up in Uganda, ART was exclusively provided by about six elite providers in Uganda since 1996 [20]. These include the Joint Clinical Research Centre (JCRC). In these handful of (predominantly) private providers, ART was almost exclusively physician-managed [20]. Nurses played supportive roles to physicians in ART principally around patient care. HIV services were not widely accessible to the majority of Ugandans who needed them due to a prohibitive fee-for-service charge that ‘crowded out’ mainstream patients. At the time, providers were able to provide physician-intensive HIV services to select Ugandans such as top government officials and high-income individuals who could afford the expensively imported anti-retroviral medicines. In June 2004, with substantial external funding from the President’s Emergency Plan for AIDS Relief (PEPFAR) and The Global Fund to Fight AIDS, Tuberculosis and Malaria, free-to-user HIV services were piloted at the national and regional referral hospitals in Uganda [20]. HIV services were subsequently decentralized to lower-level public and private health facilities across Uganda [3, 10, 21, 22]. Overnight, there was a dramatic expansion in HIV client loads. From the initial 2700 enrolled on ART in June 2004, there are currently over a million Ugandans accessing treatment [20]. The physician-intensive HIV care model could scarcely meet the continuous increase in demand for HIV treatment in Uganda. Providers resorted to non-physician cadre (such as nurses, midwives and lay workers) to meet the growing demand for ART across Uganda [3].

As the world commemorates 2020, as the International Year of the Nurse and the Midwife [20, 23], we sought to reflect on the changing roles of nurses especially with regard to the expansion in their scope of practice in HIV care and treatment in resource-limited settings. With the perspective granted by the passing of years since ART scale-up commenced in sub-Saharan Africa in 2004 [3, 10], the extension in the tasks carried out by nurses in HIV service delivery in Uganda is somewhat clearer. Although previous studies have focused on task shifting to nurses from a clinical lens of comparing patient outcomes of nurse versus physician-managed HIV care [15–19], the ways in which ART scale-up precisely transformed and re-defined the role of nurses are not well described in the current literature [23, 24]. This paper addresses this gap. We sought to describe how antiretroviral therapy scale-up in Uganda since 2004 expanded the role of nurses in HIV care and redefined what nurses are capable of accomplishing as a health worker cadre. This expansion in the roles of the nursing workforce has far-reaching impacts on health services delivery in general in the context of calls for universal health coverage (UHC) [5, 6, 23].

## Methods

### Research design

We adopted a mixed-methods sequential explanatory research design [25]. This study was conducted as part of a 4-year doctoral research project aimed at understanding the organizational strategies devised by health facilities in Uganda to promote the sustainability of anti-retroviral therapy (ART) scale-up programmes from the perspective of WHO’s building blocks of the health system framework [10, 21, 22]. The study was conducted in two phases which were implemented sequentially [26]. Phase I entailed a cross-sectional health facility survey to assess the extent to which human resource management strategies (such as task shifting) were common. Phase II entailed a multiple case study of 16 (of the 195) health facilities for an in-depth understanding of the strategies adopted and the operational context(s) underpinning these strategies [27].

### Study sites and sampling

The unit of study was health facilities in Uganda which participated in the initial phase of ART scale-up which commenced in June 2004 [3]. With regard to the health facility survey (phase I), we secured the published list of health facilities accredited to provide ART by the Ministry of Health of Uganda [28]. This published list indicates that 394 facilities were accredited to provide ART as of March 2010 and the sub-regions of Uganda where they were located. This list served as a sampling frame for our study [3]. The 394 health facilities were placed in 10 clusters based on their location in the 10 geographic sub-regions of Uganda as designated by the Uganda Bureau of Statistics [29]. Using proportionate size-to-sample technique [3], we randomly selected 195 (out of 394) health facilities [3].

In phase II, we purposively selected sixteen health facilities to achieve diversity with regard to ownership-type (public/private), setting (rural/urban/peri-urban), level of care in the Ugandan health system (tertiary/secondary) and sub-region of Uganda (e.g. Northern/Southern). Table 1 shows the characteristics of case-study facilities.

**Table 1:** Characteristics of case-study facilities

	Acronym	Ownership-type	Level of care in Ugandan health system	Setting	Geographic sub-region	Annual ART patient load (as of June 2014)
1	PUB-01	Public	Referral Hospital	Urban	South West	24,408
2	PUB-02	Public	Referral Hospital	Urban	Kampala	2408
3	PUB-03	Public	Referral Hospital	Urban	Central 2	6414
4	PUB-04	Public	District Hospital	Urban	East Central	598
5	PUB-05	Public	Health centre IV	Peri-urban	Mid-East	458
6	PUB-06	Public	Health centre IV	Rural	Mid-North	2034
7	PUB-07	Public	Health centre IV	Rural	East-Central	263
8	PUB-08	Public	Health centre IV	Peri-urban	Mid-West	298
9	PUB-09	Public	Health centre IV	Rural	North East	126
10	PNFP-01	Not for profit	Referral hospital	Urban	Kampala	4337
11	PNFP-02	Not for profit	Referral hospital	Urban	East Central	1727
12	PNFP-03	Not for profit	Health centre IV	Rural	Mid-East	647
13	PNFP-04	Not for profit	Health centre IV	Peri-urban	South West	402
14	PFP-01	For-profit	Health centre III	Urban	Mid-West	324
15	PFP-02	For-profit	Health centre II	Urban	Central 2	29
16	PFP-03	For-profit	Health centre II	Rural	Mid-North	46

### Data collection

#### Phase I

A 35-item questionnaire was self-administered by the ART clinic manager at each of the 195 health facilities. This questionnaire is described elsewhere [3, 10, 21]. A filled hard-copy questionnaire was picked on-site by a research assistant a week after it was delivered to the ART clinic manager. To mitigate potential non-response bias, phone call reminders were made to respondents after a 3-day interval [3]. The survey was conducted between January and April 2014.

#### Phase II

An interview guide was constructed with the aim of understanding the operational contexts underpinning the human resources for health strategies adopted at participating facilities. We conducted 24 face-to-face in-depth interviews (IDIs) with ART clinic managers and staff with personnel management responsibilities (such as the head of clinical services or facility in-charge) in their offices within the facilities. The interviews were conducted by the author who has an academic background in the social sciences and is experienced in qualitative research [22, 27]. The author was assisted by four research assistants with expertise in qualitative health services research. On average, these interviews lasted between 45 and 60 min. The qualitative interviews were conducted in two waves of data collection. The first one was conducted between June and August 2014 [3], and the second one was conducted between April and June 2016 [27].

### Data analysis

#### Phase I

Questionnaire data were initially entered into EpiData software (version 3.1). Data were later exported into STATA (version 13). Descriptive statistics were generated relating to the demographics of participating facilities, the extent to which varied human resources for health strategies (such as task shifting from physicians to nurses) were common.

#### Phase II

Qualitative data were analysed by thematic approach. The audio recordings of the semi-structured interviews were transcribed verbatim into text transcripts. Qualitative data analysis was carried out in four major steps [30]. However, this was a largely iterative process. The first step entailed data familiarization [30]. In this stage, the transcribed transcripts were read multiple times. In the second stage, a descriptive coding scheme was inductively generated from multiple readings of the interview transcripts. In the third stage, the emergent codes were then abstracted into thematic matrices [30]. The fourth stage involved overall interpretation and synthesis [31]. A 1-day data validation workshop was conducted involving five ART clinic managers to assess agreement in interpretation of the study findings [32]. A 1-h presentation of the emergent study findings was made by the author to five ART clinic managers to garner feedback. Their input and feedback informed the final analyses [32].

### Mixed-methods integration

The quantitative and qualitative data were merged together during the stage of overall interpretation of study findings [33]. Specifically, results of each data set were placed side by side to garner convergence in answering the study objective. The quantitative and qualitative data are presented alongside each other under the emergent sub-themes described in the “Results” section [34].

## Results

### Characteristics of participating health facilities

Overall, 195 facilities across Uganda participated in the study. With regard to ownership-type, 121 (61%) were public facilities, while 35 (18%) were private-not-for-profit and 33 (16%) were private-for-profit. HIV research clinics in our sample were 6 (3%). In Uganda, HIV research clinics are specialized health facilities primarily engaged in HIV research (such as running HIV prevention clinical trials) but with a routine service delivery arm attending to regular patients.

Table 2 shows that by level of care in the Ugandan health system, the majority of participating facilities were Health Centre IVs (sub-district facilities) at 72 (36.9%). This was followed by district hospitals at 58 (29.7%).

**Table 2:** Participating health facilities by level of care

Level of care	Number (*n*)	Percentage (%)
National referral hospital	2	0.8
Regional referral hospital	12	6.4
District hospital	58	29.7
Health centre IV (sub-district)	72	36.9
Health centre III (sub-county)	18	9.3
Health centre II ( parish)	33	16.9
Total	195	100

In terms of setting, almost half of the health facilities 88 (45%) were based in peri-urban areas (urbanized parts of rural areas). This was followed by 78 (40%) health facilities which were located in urban settings while 29 (15%) of the facilities were based in rural settings.

### The nurse in-charge: leadership roles in HIV clinics

Our cross-sectional survey reveals that across the 195 health facilities participating in the study, nurses were the most represented cadre of health workers reporting a role as ‘ART clinic manager’ or the overall leadership of HIV clinics. As Table 4 shows, 72 (36.93%) of all the 195 HIV clinics in our sample were led by nurses. Clinical officers were the second most represented cadre in the leadership of HIV clinics 66 (33.84%). In Uganda, clinical officers (COs) are a mid-level cadre of health professionals below the ladder of physicians. They attend 3 years of post-secondary training in a non-university tertiary institution [3] (Table 3).

**Table 3:** Cadre of health workers heading HIV clinics in participating facilities

Cadre	Number (out of 195)	Percentage (%)
Nurses	72	36.92
Clinical officers	66	33.84
Medical doctors	44	22.56

In the in-depth interviews with nurses, they described finding themselves in leadership positions in HIV clinics across Uganda. This was especially so in primary care-level health facilities and was certainly the case in facilities in rural settings. Rural health facilities are often shunned by more specialized health worker cadre. However, even in regional referral hospitals (such as PUB-02), the head of the stand-alone HIV clinic was a nurse.

#### Nurse-led HIV clinics in rural settings

Our survey findings reveal a rural-urban dichotomy with regard to the phenomenon of nurse-led HIV clinics. As Table 4 shows, most of the nurse-led HIV clinics were based in rural settings. However, nurse-led HIV clinics were fairly even across setting.

**Table 4:** Cadre of health workers represented in HIV clinic leadership by setting (*n* = 195)

Setting	Cadre of health worker				
	Medical doctor	Clinical officer	Nurses	Midwives	Others
Rural	06 (13.63%)	12 (18.8%)	26 (36.12%)	5	11
Peri-urban	11 (25.00%)	38 (57.58%)	23 (31.94%)	1	5
Urban	27 (61.36%)	16 (24.24%)	23 (31.94%)	2	3
Total	44 (100%)	66 (100%)	72 (100%)	8	19

Our qualitative interviews with facility in-charges shed more light on why nurse-led HIV clinics were mostly in rural settings. This was explained by a facility in-charge of one of the case-study facilities selected for in-depth understanding of the human resources for health strategies adopted by providers.

Our district being in a rural setting, those high caliber cadres like the medical officers, the pharmacists, it is a challenge to attract and retain those people. For medical officers we had a very serious gap. That’s why nurses are our main hope. (Facility in-charge, PUB-03)

Although nurse-led HIV clinics were more common in rural settings, during on-site visits to facilities participating in the national survey, it was not uncommon to find that nurses were ART clinic managers even in tertiary facilities which are predominantly in urban settings. This was certainly the case in several district hospitals we visited and even in a number of regional referral hospitals across the 10 sub-regions of Uganda we visited.

#### The management skill sets demanded by HIV clinic leadership

As overall leaders of HIV clinics, nurses reported that they were responsible for the day-to-day operational aspects of running these busy stand-alone HIV clinics. As such, they described finding themselves thrust in positions requiring generalist management knowledge and skills. Some of the management skills were identified by ART clinic managers as critical. These include competencies in human resources management such as the need to motivate over-burdened staff, ensuring sufficient stock of ART commodities including managing frequent ART stock-out events as well as resource mobilization for HIV clinics. These are areas requiring competencies that are not primarily covered during their pre-service training.

You suddenly find yourself heading the (HIV) clinic and handling crisis after crisis. Stock-outs are a chronic headache, absenteeism by some members of our staff, the CD4 machine has broken down. All these matters come to you as the sole solution provider. (ART clinic in-charge, PUB-02)

### Nurse-centred HIV care models

Findings from our national health facility survey show that in 93% (181) of the 195 health facilities, non-physicians were engaged in the clinical management of ART, including in initiating this therapy. Facility in-charges explained that they had difficulty retaining highly trained cadres such as physicians. We found that several health facilities deliberately adopted nurse-centred HIV care models as a mitigation strategy. Nurses were relied upon as the primary cadre in providing and managing ART services including initiating therapy in a number of facilities. In a large mission hospital (PNFP-01), nurses were trained to be versatile in a wide range of tasks within the HIV clinic. This was described by the ART programme manager of one of the mission hospitals as follows:

I think my nurses are the best asset I have here. HIV has brought out the potential of nurses. We used to think ‘oh, she is just a nurse’. But they can do a lot! And I think that’s our strength here. They will retrieve records and patient files. They will manage the reception. They will dispense. They can do a lot. And I think that’s our strength here. (ART program manager, PNFP-01)

To enable nurses to broaden and widen their competencies, they were continuously trained through weekly in-house ‘continuing medical education’ on-site trainings to manage an enormous expansion in their traditional scope of practice. Mentorships and on-site support supervision of nurses in HIV care were raised as strategies for unlocking the potential of the nursing workforce. Off-site trainings in HIV care and treatment were cited as another enabler in the expansion of the scope of practice of the nursing workforce at participating health facilities. Several of the off-site trainings were said to be funded by donors such as PEPFAR- subsidiaries while others were said to be organized by Uganda’s Ministry of Health.

We do a lot of in-house staff development. We know that every Wednesday is a CME (continuing medical education) day. We conduct on-site training for our nurses… many never did presentations but now our nurses do. They can stand up and do presentations, I mean power-point. So we empower them because we don’t pay them that much and when there is an off-site workshop we try to make sure that everyone gets a chance to go to. (ART clinic manager, PNFP-02)

Across our interviews with facility in-charges, there was a widely held perception that nurses were more stable at health facilities compared to cadres with longer post-secondary school training. Physicians were perceived to be mobile on account of their affinity for accepting additional training opportunities and better job offers. As such, nurses were perceived as a cadre that were dependable and could be relied on for long-term planning due to their tendency to remain stable in the same employment and work stations compared to more specialized cadres.

We focus on especially nurses because those tend to stick around for years unlike young doctors who frequently opt for further training and leave when better opportunities arise. (Head of clinical services, PNFP-01)

Due to the low pay common in public facilities in Uganda, nurses were perceived to be more resilient.

We have some cadres who are not easy to work with due to our low wages. For instance, pharmacists, because they have so many (private) pharmacies around which they are supervising and they are crying that the pay is low. So, they always want to get something from outside. So, those ones have been a problem for us but not nurses and midwives. Another cadre is the medical doctors also. They have been a problem to us because most of them were crying of the low salary. (Hospital administrator, PUB-01)

#### Nurse-led HIV care

Although a number of health facilities indicated that their HIV service delivery was *nurse-centred*, 38 of the 195 (18%) health facilities in our survey indicated that they adopted *nurse-led* care models. Under this model, nurses led service delivery along the entire HIV care continuum including in initiating ART. Nurses were trained to have a broad diversity of competencies along the HIV care continuum. The nature of tasks assigned to nurses ranged from tuberculosis (TB) management to counselling of patients as described below:

Every nurse here is a dispenser, a counselor, is a triage nurse, she can work in the laboratory and at least can do phlebotomy and at least make an HIV test and work in a treatment room and work in a TB room. So there is a lot of task shifting and multi-tasking so my nurses can do (male medical) circumcision, where I can’t. (ART clinic manager, PUB-04)

### Differentiated task-shifting to nurses

In a section of tertiary facilities (e.g. PNFP-01, PUB-02, PUB-03), we found a more differentiated approach to task shifting to nurses. This was in contrast to the practice in some health facilities of devolving clinical management of ART from physicians to nurses.

#### Stable patients managed by nurses

Patients with advanced HIV disease were reported to be managed by clinician cadres such as clinical officers and physicians while patients who were deemed stable on ART were handled by nurses.

The patient numbers are big but we have trained our nurses. Our nurses help us to handle the stable clients. Nurses can help a great deal because patients need to see a doctor at least once or twice a year. (Clinician, PNFP-01)

#### Nurse-led community-based outreaches

Nurses were often assigned to lead HIV care outreach teams in communities. A mission hospital (PNFP-01) in our case-study facilities run a home-based HIV care programme for stable patients who live within a 5-km radius of the hospital. Nurses often led typically four-member teams that also comprised ‘expert patients’. They conducted reviews during these home visits and distributed ART refills. The nurse-led community outreach programmes were aimed at decongesting overcrowded HIV clinics and easing transport burdens on patients. The nursing workforce was reported to be critical in these endeavours.

## Discussion

The changing roles and increasing importance of the nursing workforce in health service delivery in resource-limited settings are the areas that are not adequately documented and sufficiently recognized in the current literature [6, 23, 24]. We set out to describe how the role of nurses in health facilities in Uganda expanded tremendously in Uganda during the era of ART scale-up that commenced in 2004 [10]. We found that nurses were the most represented cadre of health workers involved in the overall leadership of HIV clinics across Uganda. Most of the nurse-led HIV clinics were based in rural settings although this trend was fairly even across the rural and urban divide. A number of health facilities in our sample deliberately adopted nurse-centred HIV care models owing to an inability to attract and retain cadres with longer training cycles. Several health facilities devised differentiated models of task shifting from physicians to nurses in which the latter handled patients who were stable on ART. Overall, our study reveals a wide expansion in the scope-of-practice of nurses. Nurses were thrust in roles of HIV disease management that were traditionally the preserve of physicians. In many health facilities, the nursing cadre were the backbone of HIV service delivery. These expanded roles include prescribing ART, tuberculosis (TB) management and ART adherence counselling.

An important contribution of this paper is in providing quantitative data showing the extent of representation of nurses in the overall leadership of HIV clinics in a national sample of health facilities in Uganda. In this study, we found that nurses were the most represented cadre of health workers indicating a role as ‘ART clinic manager’. Due to the leadership and management roles associated with leading HIV clinics, management generalist skill sets became imperative. Nurses frequently grappled with the challenge of motivating personnel in the face of rapidly expanded workloads owing to escalating HIV client loads. Nurses had to frequently ponder responses to chronic ART medicines stock-outs and to manage associated supply chains. Our study therefore suggests that leadership and management training needs to be strengthened in nurse pre-service training curricula in Uganda and other resource-limited settings to prepare them for the leadership roles that many nurses are frequently thrust into [6, 35, 36]. Importantly, our study highlights the importance of reforming regulatory frameworks governing nursing workforce scope of practice in Uganda and other countries.

Although our data were collected between 2014 and 2016, studies published after this period strongly suggest that task shifting from physicians to nurses in HIV care is still common in health facilities in Uganda. Hence, our study findings still resonate with current practice [12, 37–43]. These data and subsequent published studies point to a mounting evidence base supporting an expanded role for nurses and other non-physician cadre in the quest to accelerate progress towards UNAIDS’s 95-95-95 targets and in advancing the universal health coverage (UHC) agenda [5, 23, 44].

Our survey results show that most of the nurse-led HIV clinics were based in rural areas. These data provide further empirical credence to notions of the mal-distribution of health workers in sub-Saharan Africa [1, 5] which suggests that more specialized cadres such as physicians and pharmacists shun rural settings. Studies have highlighted how frequently nurses are the only cadre available to provide health care in rural, remote outposts or hard-to-reach areas [1, 2, 6]. These findings suggest that in rural settings, HIV care is largely nurse-led. There is therefore a need to strengthen HIV disease management in nurse education curricula as the likelihood of nurses engaging in the clinical management of ART is high. Given that nurses are the mainstay of health service delivery in rural settings, their shortage per capita is a fundamental constraint that merits governance reforms [45]. To address the underproduction of nurses in Uganda, there have been calls for the promotion of science education in secondary schools to boost the pool of prospective nurse recruits [5].

Beyond the global HIV response, Rabkin and colleagues [46] have noted the need to leverage HIV lessons such as task shifting to nurses in the response to the burgeoning non-communicable diseases’ (NCDs) epidemic if given the requisite training, standardization of guidelines and mentorship support.

We found that several health facilities had deliberately adopted *nurse-led* HIV care models. Nurses were empowered to be multi-skilled with a wide range of competencies across the continuum of HIV care right from HIV testing to mainstream clinical HIV disease management. Our findings underscore the need for regular refresher trainings in HIV care and treatment for nurses and other less-specialized cadre who are the mainstay of ART service delivery across Uganda. This could be in the form of regular seminars and workshops whether these be in-house or off-site by the Ministry of Health and donors. On-site support supervision and mentorships of nurses by specialized cadre have been acknowledged in the literature as supporting interventions [3, 12, 13, 47]. Although our study reveals that 93% of the 195 health facilities in Uganda allowed non-physician cadre to prescribe ART, Baine et al. [12] and Dambisya et al. [13] have observed the lack of a formal policy on task shifting in Uganda. Such an enabling policy would permit advanced roles for nurses in HIV care and treatment and in other disease responses [36, 48]. A recent multi-country study by Ford and colleagues [47] found that, in many countries, policies currently provide for ART initiation on first-line ART by non-physician cadre although the same guidelines preclude them from managing children and patients on second-line ART. This is  inspite of an estimated 3 million people in sub-Saharan Africa with an unmet need of second-line ART [47]. Our study findings add to calls for reforms in regulatory frameworks that acknowledge the proven competence of nurses and allow for advanced roles for nurses in their permitted scope of practice [23, 24, 36, 48, 49].

On the other hand, our study reveals that select health facilities adopted differentiated models of task shifting to nurses. Whereas patients with advanced HIV disease were managed by the clinician cadre (especially physicians), patients who were stable on ART were managed by nurses. In a number of health facilities running community-based platforms of HIV care such as home-based visits, the monitoring of patients within communities was nurse-led. Our findings find resonance with the currently topical Differentiated Service Delivery (DSD) agenda [50–52]. DSD calls for tailoring HIV care to the needs of individual patients including differentiating the cadre assigned to manage patients based on clinical criteria such as those who are stable and those who are not [9, 50]. The role of nurses in managing less-intensive models of HIV care for stable patients especially those enrolled in community-based ones such as community outreach ART refill pick-up points and community-based patient adherence support groups is especially timely [9, 10, 50–52].

## Conclusion

Overall, our study reveals a wide expansion in the scope of practice of nurses during the era of ART scale-up in Uganda. Nurses were thrust in roles of HIV disease management that were traditionally the preserve of physicians. Our study highlights the importance of reforming regulatory frameworks governing nursing workforce scope of practice in Uganda such as the need for developing a policy on task shifting which is currently lacking in Uganda.

## Data Availability

The datasets generated during and/or analysed during the current study are not publicly available due to ethical reasons but are available from the corresponding author on reasonable request.

## Abbreviations

AIDS: Acquired immune deficiency syndrome
ART: Anti-retroviral therapy
ARVs: Anti-retrovirals
HRH: Human resource for health
NIMART: Nurse Initiated and Managed Anti-retroviral therapy
PEPFAR: The Presidents’ Emergency Plan for AIDS Relief
SSA: Sub-Saharan Africa
WHO: World Health Organization
